# Comparative Characterization and Risk Stratification of Asymptomatic and Presymptomatic Patients With COVID-19

**DOI:** 10.3389/fimmu.2021.700449

**Published:** 2021-07-09

**Authors:** Lei Shi, Rong Ding, Tingting Zhang, Wei Wu, Ziyu Wang, Xiuzhi Jia, Kening Li, Yuan Liang, Jie Li, Mengyan Zhu, Bin Huang, Lingxiang Wu, Min Wu, Jing Chen, Chaochen Wang, Bin Huang, Caidong Liu, Hongbing Shen, Qianghu Wang, Xinyi Xia, Pengping Li, Sali Lyu, Ying Xiao

**Affiliations:** ^1^ Affiliated First People’s Hospital of Kunshan, Gusu College of Nanjing Medical University, Suzhou, China; ^2^ Center for Global Health, School of Public Health, Nanjing Medical University, Nanjing, China; ^3^ Department of Bioinformatics, Nanjing Medical University, Nanjing, China; ^4^ School of Biological Science and Medical Engineering, Southeast University, Nanjing, China; ^5^ Central Lab of Biomedical Research Center, Sir Run Run Shaw Hospital, School of Medicine, Zhejiang University, Hangzhou, China; ^6^ Department of Breast Surgery, The Second Affiliated Hospital, Zhejiang University School of Medicine, Zhejiang University, Hangzhou, China; ^7^ ZJU-UoE Institute, Zhejiang University School of Medicine, Zhejiang University, Haining, China; ^8^ The Comprehensive Cancer Center of Nanjing Drum Tower Hospital, The Affiliated Hospital of Nanjing University Medical School & Clinical Cancer Institute of Nanjing University, Nanjing, China; ^9^ Department of Laboratory Medicine, Nanjing First Hospital, Nanjing Medical University, Nanjing, China; ^10^ Jiangsu Key Lab of Cancer Biomarkers, Prevention and Treatment, Collaborative Innovation Center for Personalized Cancer Medicine, Nanjing Medical University, Nanjing, China; ^11^ Collaborative Innovation Center for Cardiovascular Disease Translational Medicine, Nanjing, China; ^12^ COVID-19 Research Center, Institute of Laboratory Medicine, Jinling Hospital, Nanjing University School of Medicine, Nanjing Clinical College of Southern Medical University, Nanjing, China; ^13^ Joint Expert Group for COVID-19, Department of Laboratory Medicine & Blood Transfusion, Wuhan Huoshenshan Hospital, Wuhan, China

**Keywords:** risk stratification, COVID-19, asymptomatic patients, presymptomatic patients, T cell exhaustion

## Abstract

The identification of asymptomatic, non-severe presymptomatic, and severe presymptomatic coronavirus disease 2019 (COVID-19) in patients may help optimize risk-stratified clinical management and improve prognosis. This single-center case series from Wuhan Huoshenshan Hospital, China, included 2,980 patients with COVID-19 who were hospitalized between February 4, 2020 and April 10, 2020. Patients were diagnosed as asymptomatic (n = 39), presymptomatic (n = 34), and symptomatic (n = 2,907) upon admission. This study provided an overview of asymptomatic, presymptomatic, and symptomatic COVID-19 patients, including detection, demographics, clinical characteristics, and outcomes. Upon admission, there was no significant difference in clinical symptoms and CT image between asymptomatic and presymptomatic patients for diagnosis reference. The mean area under the receiver operating characteristic curve (AUC) of the differential diagnosis model to discriminate presymptomatic patients from asymptomatic patients was 0.89 (95% CI, 0.81-0.98). Importantly, the severe and non-severe presymptomatic patients can be further stratified (AUC = 0.82). In conclusion, the two-step risk-stratification model based on 10 laboratory indicators can distinguish among asymptomatic, severe presymptomatic, and non-severe presymptomatic COVID-19 patients on admission. Moreover, single-cell data analyses revealed that the CD8+T cell exhaustion correlated to the progression of COVID-19.

## Introduction

Coronavirus disease 2019 (COVID-19) is a self-limiting disease in more than 80% of patients, and severe pneumonia occurs in approximately 15% of patients. However, rapid disease transmission turned the COVID-19 outbreak into a pandemic (1–3). The COVID-19 outbreak began and spread worldwide in December 2019 (4, 5), and on April 5, 2021, the World Health Organization reported a total of 131,020,967 COVID-19 cases globally.

Epidemiological estimates suggest that 50% of patients testing positive for severe acute respiratory syndrome coronavirus 2 (SARS-CoV-2) on nucleic acid testing have no typical symptoms (i.e., fever, cough, or shortness of breath) (6–9). Asymptomatic and presymptomatic viral shedding not only poses a big challenge to infection control but also adds complexity to appropriate clinical decision-making and resource allocation (10, 11). It is noteworthy that the mortality rate varies from country to country, possibly reflecting the differences in how rapidly local health authorities respond to isolate and initiate effective stratification and management strategies for the infected population (12). Hospitalization of all infected persons places too much burden on the healthcare system, but management based only on symptoms may not be appropriate (13). The prediction of asymptomatic, non-severe presymptomatic, and severe presymptomatic COVID-19 in patients could facilitate clinical resource allocation by health authorities, and improve the prognosis of patients. However, at present, there is no method for the risk stratification of asymptomatic and presymptomatic COVID-19 patients.

This study was conducted to comparatively evaluate the clinical characteristics of COVID-19 patients and construct a two-step risk-stratification model based on clinical indicators to distinguish among asymptomatic, severe presymptomatic, and non-severe presymptomatic COVID-19 patients on admission.

## Materials and Methods

### Data Collection

From February 4, 2020 to April 10, 2020, a total of 3,046 COVID-19 patients’ data was collected from Wuhan Huoshenshan Hospital in China. The extracted information from electronic medical records included clinical diagnosis, viral RNA load, chest CT imaging, and laboratory indicators. This study was approved by the hospital ethics committee, and the written informed consent was provided by every patient enrolled.

### Patients Classification

Patients were classified into asymptomatic, presymptomatic, and symptomatic groups based on the retrospective review of the medical records. Asymptomatic COVID-19 patients were those with SARS-CoV-2 nucleic acid test positive but without any clinical symptom or only with stable pre-exist chronic symptoms (e.g., chronic cough without worsening). Presymptomatic patients were those asymptomatic inpatients on admission but then developed typical (fever, cough, shortness of breath) or atypical symptom (14, 15). Symptomatic patients were those who had typical clinical symptoms on admission. Clinical characteristics of patients among asymptomatic, presymptomatic and symptomatic COVID-19 were shown in **Table S1**.

The diagnosis and severity degree of each patient was determined according to the clinical classification criterion in Diagnosis and Treatment Protocol for Novel Coronavirus Pneumonia released by the National Health Commission (6th or 7th edition). Patients were classified into mild or moderate, severe, and critical cases, depending on the clinical symptoms, radiological characteristics, and the amount of oxygen requirements. Mild patients had mild clinical symptoms and no imaging sign of pneumonia. Moderate patients had fever and respiratory symptoms with radiological findings of pneumonia. Severe patients met any of the following criteria: (1) Respiratory distress (≧30 breaths/min); (2) Oxygen saturation ≤ 93% at rest; (3) Arterial partial pressure of oxygen (PaO2)/fraction of inspired oxygen (FiO2) ≦ 300 mmHg. Critical patients were those with respiratory failure requiring mechanical ventilation, shock, or requiring ICU care with other organ failure (16). In our analysis, the clinically defined severe or critical COVID-19 patients were grouped into severe cases group, and the clinically defined mild or moderate COVID-19 patients were defined as non-severe group cases.

### Baseline Correction and Statistical Analysis

The propensity score matching (PSM) method was used to screen symptomatic patients based on the clinical profiles of asymptomatic and presymptomatic COVID-19 patients upon admission to hospital (17). Confounding factors include age, gender, hypertension, diabetes, cardiovascular disease, cerebrovascular disease, cancer, chronic obstructive pulmonary disease, chronic kidney disease, chronic liver disease, and immunodeficiency. For continuous variables, the median and interquartile range (IQR) were used to show the distribution, and the Wilcoxon rank-sum test with two-sided was used to compare the differences between groups. Number and percentage were used to show discrete variables, and Fisher’s exact test with two-sided was used to compare the differences. All statistical analyses were performed using R software.

### Single-Cell Transcriptional Profiles of COVID-19 Patients

Single-cell sequencing data of eight healthy lung tissues were downloaded from the GEO database with the accession number GSE122960 (18), data of nine bronchoalveolar lavage fluid (BALF) samples from three mild, and six severe COVID-19 patients were downloaded from the GEO database with the accession number GSE145926 (19). The quality control, standardization, integration, dimensionality reduction, and unsupervised cell clustering of the samples were carried out using R package Seurat (v3.0.2). For each patient, the same standard for quality control was used: 1) each cell expressed 200-6,000 genes, and unique molecular identifiers (UMIs) must be greater than 1,000; 2) each gene was expressed in at least three cells; 3) The proportion of mitochondrial genes was less than 10%.

### Two-Step Risk Stratification Model

In order to improve prognosis of patients without clinical symptoms on admission, we designed a two-step risk-stratification model. (1) Initially, we constructed a random forest classifier based on AdaBoost with 39 asymptomatic and 34 presymptomatic patients. The diagnostic model could accurately classify asymptomatic and presymptomatic patients in 5-fold cross-validation. (2) Next, to predict the progression of presymptomatic COVID-19 patients, we constructed a random forest classifier based on AdaBoost with the training set of 1,752 symptomatic patients. The model was then validated in 9 non- severe and 25 severe presymptomatic patients.

The r-adabag package was used to perform the ensemble learning. Candidate features used to construct the classifier were 63 laboratory indicators indicated in **Table S2** (comparison among asymptomatic, presymptomatic and symptomatic COVID-19 patients), **Table S3** (comparison between non-severe and severe COVID-19 patients). Patients whose laboratory tests with more than 80% missing value were excluded. To apply the model for early application and avoid the interference of therapy, we only selected the laboratory test data within one day after admission. If the patient had multiple tests, the mean value was used for analysis. Missing values were supplied by the method of Conditional Mean Completer (CMC).

## Results

### Demographic and Clinical Characteristics of Asymptomatic, Presymptomatic, and Symptomatic COVID-19 Patients

**Figure 1:**
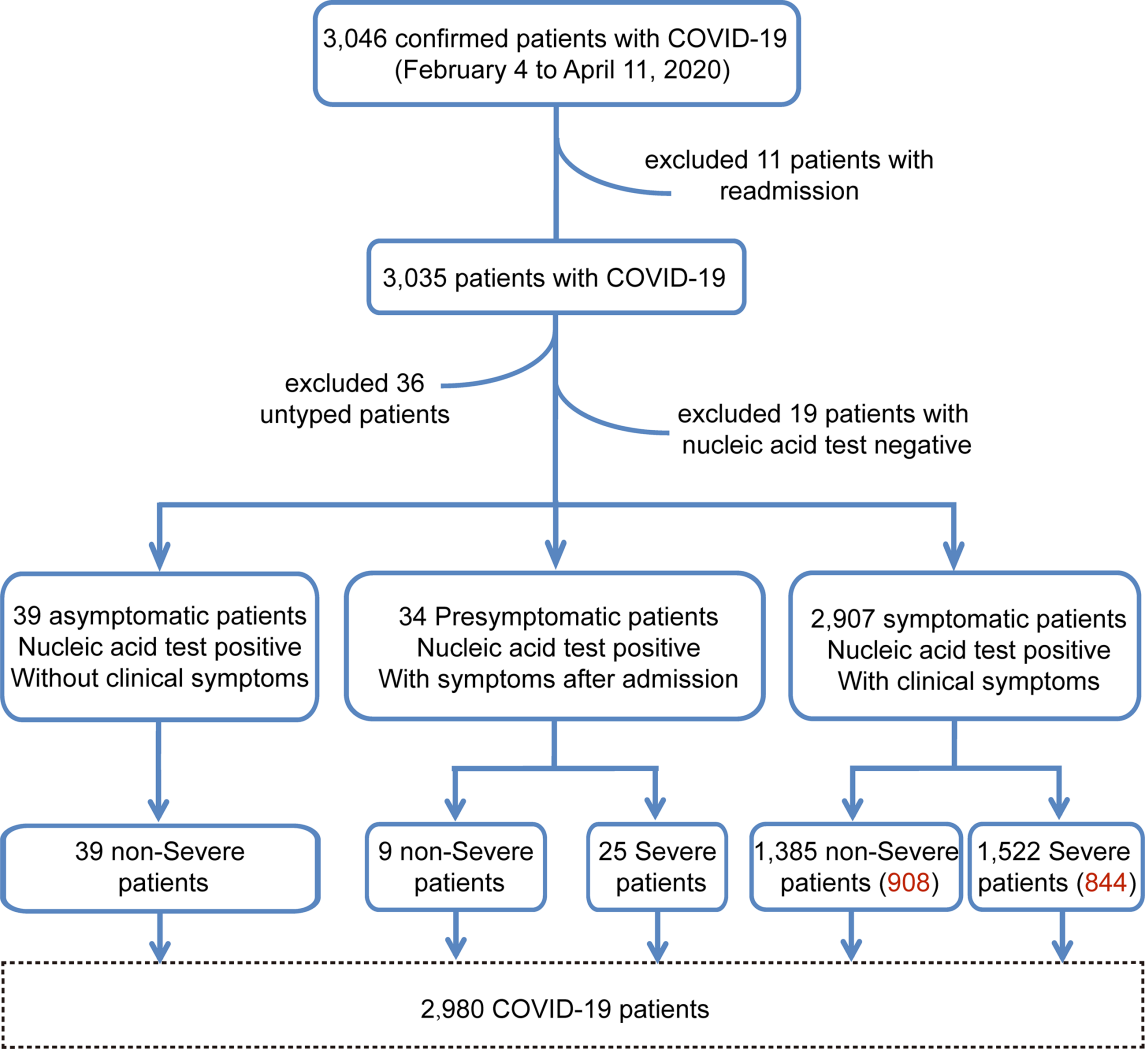
The enrollment workflow of the COVID-19 cohort. The workflow chart shows the screening process of the cohort samples. The number of red words in parentheses represents the sample size after baseline correction.

Of the initial 3,046 COVID-19 patients who were screened for study inclusion, 66 were excluded, including those who were readmitted (n=11), had negative nucleic acid test results (n=19), or had missing data on the degree of illness severity (n=36). Thus, 2,980 patients were included in the study: 39, 34, and 2,907 asymptomatic, presymptomatic (non-severe, n=9, 26%; severe, n=25, 74%), and symptomatic (non-severe, n=1,385, 48%; severe, n=1,522, 52%) patients, respectively (**Figure 1**).

**Figure 2:**
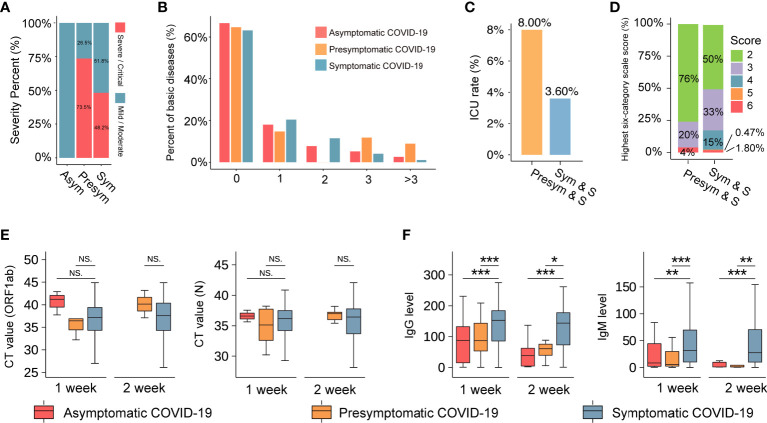
The basic information of the COVID-19 cohort. **(A)** The severity percentage of asymptomatic, presymptomatic, and symptomatic patients with COVID-19. Blue represents non-severe (mild or moderate), and red represents severe or critical. **(B)** The comorbidity rate of patients with COVID-19. The vertical axis represents the percentage of comorbidities (%). The horizontal axis represents the number of comorbidities one patient has. **(C)** A comparison of the ICU rate between patients with presymptomatic and symptomatic severe COVID-19. The vertical axis indicates the rate of ICU. ‘Pre-sym & S’ and ‘Sym & S’ represent the severe presymptomatic and symptomatic patient group, respectively. **(D)** The highest six-category scale scores in severe COVID-19 patients. The vertical axis indicates the number of highest six-category scale scores (%). Scores represent their severity, including 1-discharged, 2-hospitalization without requiring oxygen therapy, 3-hospitalized with low-flow oxygen therapy, 4-hospitalized with non-invasive ventilation and/or high-flow oxygen therapy, 5-hospitalized for ECMO and/or mechanical ventilation, and 6-death. **(E)** and **(F)** The boxplot reflects the viral load (CT value) and SARS-COV-2 antibody IgG and IgM levels in three groups at 1 week or 2 weeks after admission. *** represents *P*-value < 0.001. ** represents *P*-value < 0.01. * represents *P*-value < 0.05. NS. represents *P*-value > 0.05. The three colors of red, orange and blue represent the asymptomatic, presymptomatic, and symptomatic group, respectively.

To ensure the even distribution of confounders among the study groups, baseline correction was performed (17), and we obtained a sample of 1,752 symptomatic COVID-19 patients, of whom 908 (51.8%) and 844 (48.2%) had non-severe and severe illness, respectively (**Figure 2A**). The commonest symptoms in symptomatic patients from disease onset to hospital admission were fever (n=2,178, 75%) and cough (n=2,097, 72%). Shortness of breath, respiratory distress, and fatigue were observed in 1,320 (45%), 1,157 (40%), and 681 (23%) patients, respectively. Less common symptoms in symptomatic COVID-19 patients included muscle soreness (n=118, 4%), diarrhea (n=66, 2%), headache (n=18, 1%), dizziness (n=26, 1%), nausea (n=21, 1%), and vomiting (n=21, 1%). Of note, respiratory failure, a sign of severe illness, was observed in only 36 symptomatic COVID-19 patients with pneumonia (<1%; **Table S1**).

The median age of the patients was 60 years (IQR: 50–68 years), and there were no significant differences in age and sex among the three groups. At least two comorbidities (such as hypertension and diabetes) were detected in asymptomatic (15.38%), presymptomatic (20.58%), and symptomatic (16.44%) COVID-19 patients (**Figure 2B**). Surprisingly, those in the presymptomatic severe group had higher rates of admission to the intensive care unit (ICU; 8.0% *vs*. 3.6%, P-value=0.24, **Figure 2C**) and mortality (4% *vs*. 1.8%, P-value=0.37, **Figure 2D**) than patients in the symptomatic severe group. Additionally, there were no significant differences in viral RNA load among asymptomatic, presymptomatic, and symptomatic patients (**Figure 2E**) at 1 week or 2 weeks after admission. It indicated that infected persons may still transmit the virus to others and should be quarantined, regardless of symptoms (6). However, at these time points, the levels of specific immunoglobulin G (IgG) and immunoglobulin M (IgM) antibodies against SARS-CoV-2 antigens containing nucleocapsid (N) and spike (S) protein were significantly higher in symptomatic patients than in asymptomatic and presymptomatic patients (**Figure 2F**), indicating that patients might have variable antibody reactions despite similar viral RNA loads.

### Laboratory Indicators in Asymptomatic and Presymptomatic COVID-19 Patients

**Figure 3:**
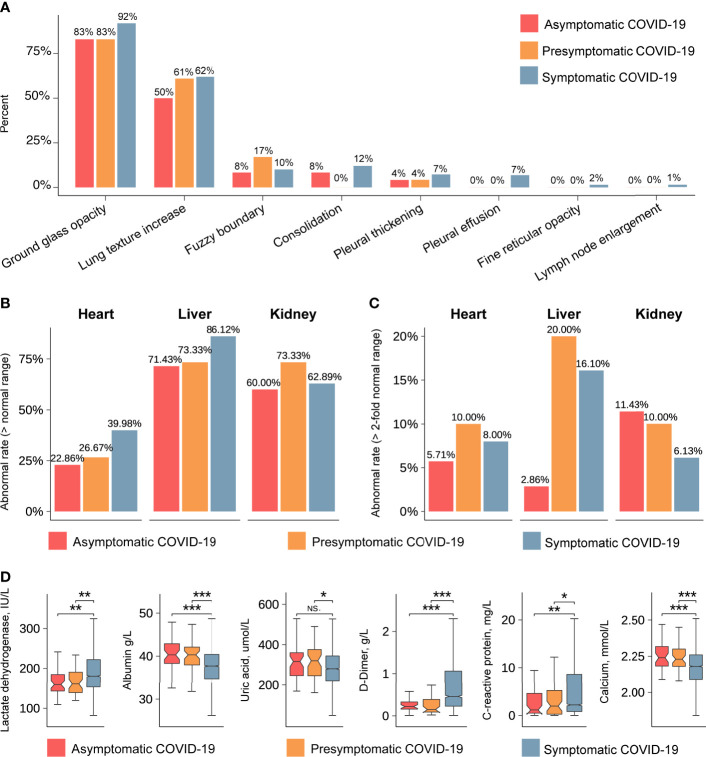
Radiological characteristics and laboratory indicators detected in asymptomatic, presymptomatic, and symptomatic patients. **(A)** The percentages of typical characteristics in chest CT imaging. The vertical axis indicates the percentages of typical characteristics. The horizontal axis represents the typical characteristics of the lung. Red, orange and blue represent the asymptomatic, presymptomatic, and symptomatic group, respectively. **(B)** The abnormal rate of organs, including heart, liver, and kidney (> normal range). The vertical axis indicates the rate of abnormal. **(C)** The abnormal rate of organs, including heart, liver, and kidney (>2-fold normal range). **(D)** The values of laboratory indicators after admission. The vertical axis indicates the values of laboratory indicators. The *P*-value was calculated using the Wilcoxon’s rank-sum test and shown at the top of each panel. Boxplots represent the 25th and 75th percentiles, with midlines indicating the median values. *** represents *P*-value < 0.001. ** represents *P*-value < 0.01. * represents *P*-value < 0.05. NS. represents *P*-value > 0.05.

On admission, the most common computed tomography (CT) findings were ground-glass opacity and lung-texture increase in asymptomatic (83% and 50%, respectively), presymptomatic (83% and 61%, respectively), and symptomatic (92% and 62%, respectively) patients (**Figure 3A**), with no significant intergroup differences (20). Other chest CT characteristics such as fuzzy boundary (range 8–17%), consolidation (0–12%), and pleural thickening (range 4–7%) were observed in asymptomatic, presymptomatic, and symptomatic patients, but without statistical significance. Interestingly, pleural effusion (7%), fine reticular opacity (2%), and lymph node enlargement (1%) were only observed in symptomatic patients. The presence of enlarged lymph nodes in symptomatic patients may indicate a stronger immune response.

To obtain more information on the affected organs, we compared laboratory indicators between the groups of asymptomatic, presymptomatic, and symptomatic COVID-19 patients. The rates of abnormal (out of the reference range) laboratory indicators associated with liver and kidney function were all above 60% in each group (**Figure 3B**), and the rates of abnormal laboratory indicators associated with heart function were less than 40%. Specifically, when the threshold for abnormality was set to two-fold points from the reference range, asymptomatic patients showed lower rates of abnormal laboratory indicators of hepatic function (2.8% *vs* 20%, *P*-value < 0.05) and cardiac function (5.7% *vs* 10%, *P*-value > 0.05) and comparable rates in terms of kidney function (11.43% *vs* 10%, *P*-value > 0.05, **Figure 3C**) than presymptomatic patients. Abnormal levels of lactate dehydrogenase, albumin, uric acid, D-dimer (DD), C-reactive protein, and calcium were less frequently observed in asymptomatic and presymptomatic patients than in symptomatic patients (**Figure 3D**, **Table S2** and **Table S3**). These analyses indicate that the liver and kidney were the most common affected organs in all COVID-19 patients, whereas indicators for heart and liver function showed the most significant differences between asymptomatic and presymptomatic patients.

### Diagnosis and Prognosis Stratification Model for Asymptomatic and Presymptomatic Patients

**Figure 4:**
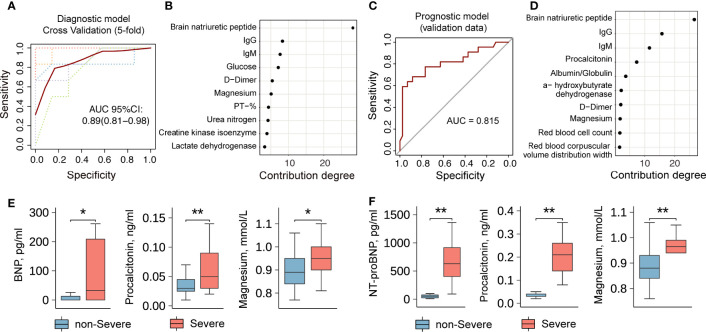
A diagnosis and severity prediction model for COVID-19 patients. **(A)** The diagnostic model for asymptomatic and presymptomatic patients. Lines represent the Receiver Operating Characteristic (ROC) curve of diagnostics of training (red line) and five-fold cross-validation (dotted lines). The dotted lines in different colors represent the results of 5-fold cross-validation. **(B)** The contribution degrees of markers (Top 10) in the diagnostic model. The horizontal axis represents the contribution degree of laboratory indicators. **(C)** The prognostic model for the severity of presymptomatic patients. **(D)** The contribution degrees of markers (Top 10) in the prognostic model. The horizontal axis represents the contribution degree of laboratory indicators. **(E)** and **(F)** A comparison of laboratory indicators between non-severe and severe patients without symptom on admission. The data set in **(E)** comes from Wuhan Huoshenshan Hospital in China. The validation data set in **(F)** comes from the First People’s Hospital of Jiangxia District of Wuhan, Hubei, China. ** represents *P*-value < 0.01. * represents *P*-value < 0.05.

To ascertain the differences between severe and non-severe COVID-19 patients who were asymptomatic at the time of admission, data of the 63 indicators listed in **Table S2** and **Table S3** were retrieved and systematically compared. We found systematic differences in laboratory indicators between severe and non-severe COVID-19 patients who were asymptomatic on admission (**Figure S1** and **Table S3**). To optimize precise risk-stratification management and improve the prognosis of patients, we constructed a two-step risk-stratification model with the data obtained from 39 asymptomatic, 34 presymptomatic, and 1,752 symptomatic COVID-19 patients. The initial diagnostic model developed with the top 10 laboratory indicators could accurately classify asymptomatic and presymptomatic patients (area under the curve [AUC]=0.89, 5-fold cross-validation 95% confidence interval [CI] 0.81–0.98; **Figure 4A**), and the degrees of contribution of the relevant 10 indicators are shown in **Figure 4B**. It is noteworthy that the top five indicators (brain natriuretic peptide (BNP), IgG, IgM, glucose (GLU), and DD) might have similar stratification efficiencies (AUC=0.86, 5-fold cross-validation 95% CI, 0.73–1.00, **Figure S2A**).

To stratify the non-severe and severe presymptomatic patients, we included the non-severe and severe symptomatic patients in a training dataset, and the constructed model with the top 10 laboratory indicators was then used to predict the severity of disease in presymptomatic patients with an AUC of 0.82 (**Figure 4C**). The degrees of contribution of the 10 indicators included in this model are shown in **Figure 4D**. The top five indicators were BNP, IgG, IgM, procalcitonin (PCT), and albumin/globulin ratio (A/G ratio), which could accurately predict illness severity (AUC=0.75, **Figure S2B**), and their degrees of contribution were 26%, 16%, 11%, 7%, and 4%, respectively (**Figure 4D**). Severe presymptomatic patients had significantly higher levels of BNP, PCT, magnesium, etc. than non-severe presymptomatic patients (**Figure 4E**). Moreover, we obtained the validation data of 27 presymptomatic patients (non-severe, n=25; severe, n=2) from another study center (the First People’s Hospital, Jiangxia District, Wuhan, China) and found that the levels of N-Terminal-proBNP (NT-proBNP), PCT, magnesium, etc., were high in severely ill COVID-19 patients (**Figure 4F**). These results suggest that the application of laboratory indicators to stratify asymptomatic and presymptomatic COVID-19 patients is feasible, and such a risk-stratification model could be used to screen non-severe and severe presymptomatic patients on admission. The package-based stratification calculator was provided online (https://github.com/liangyuan-njmu/SeverePredictModel) for clinicians to assess whether presymptomatic patients will show disease progression.

### CD8+ T Cell Exhaustion Correlated to the Progression of COVID-19

**Figure 5:**
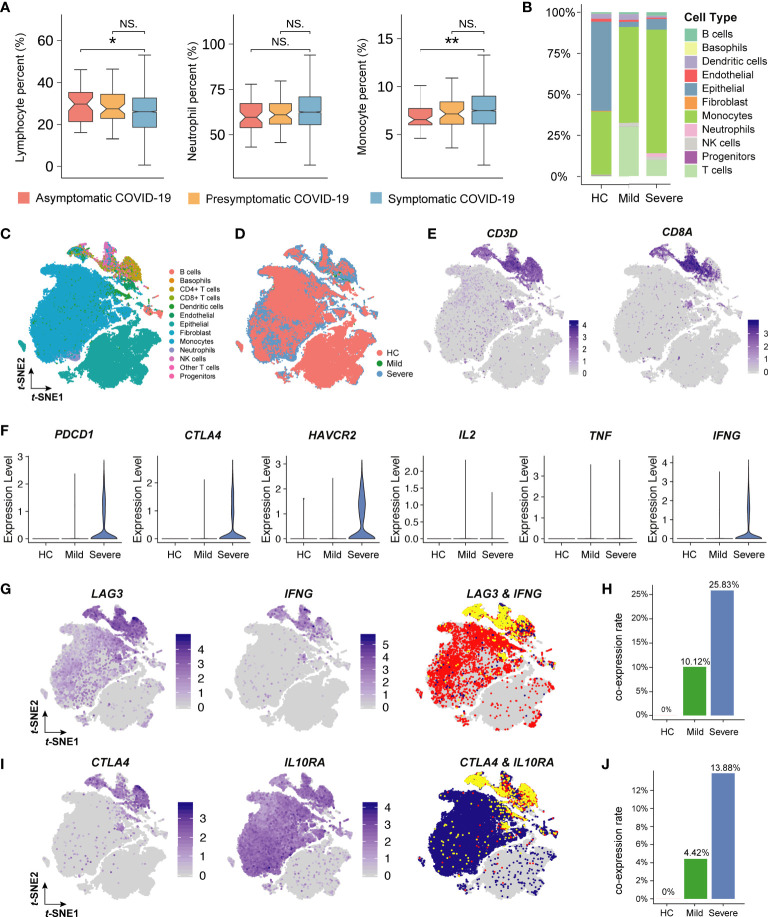
Single-cell analysis of immune cells in COVID-19 patients. **(A)** The box plot describes the proportion of lymphocytes, neutrophil, and monocyte. The vertical axis indicates the values of laboratory indicators. The *P*-value was calculated using Wilcoxon’s rank-sum test and shown at the top of each panel. Boxplots represent the 25th and 75th percentiles, with midlines indicating the median values. ** represents *P*-value < 0.01. * represents *P*-value < 0.05. NS. represents *P*-value > 0.05. **(B)** The histogram shows the proportion of 11 main cell types (including 13 clusters, 3 T cell subpopulations are merged as one) in healthy people (HC), mild patients, and severe patients. The vertical axis indicates the fraction of the cell type. Each color represents a cell type. **(C)** The t-distributed stochastic neighbor embedding (t-SNE) plot of 13 cell clusters for seventeen patients with or without COVID-19. **(D)** The t-SNE plot of 13 cell clusters colored by patient type. Red, green, and blue represents the HC, mild, and severe group, respectively. **(E)** The t-SNE plot of CD+8 T cells colored by cell-type-specific markers. The shade of the color represents the level of gene expression. **(F)** Violin plots of the inhibitory-receptors and cytokines’ gene expression. The vertical axis indicates the level of gene expression. **(G)** and **(I)** The t-SNE plot of inhibitory-receptor coexpression patterns. Yellow represents the cells expressing two genes at the same time. **(H)** and **(J)** The bar plot shows the proportion of inhibitory-receptor coexpression patterns. The vertical axis indicates the rate of coexpression patterns.

Lymphocytopenia, defined as a lymphocyte count of < 1.5x10^9^/L, was seen in symptomatic patients (35%) but was less frequently observed in asymptomatic (14%) and presymptomatic patients (17%). There were significant differences in lymphocyte percentages between asymptomatic and symptomatic patients (29.7% *vs.* 26.1%, *P*-value < 0.05, **Figure 5A**). Moreover, we analyzed the expression levels of cytokines directly from plasma without stimulation in symptomatic COVID-19 patients and found that the expression levels of interleukin 6 (IL-6), IL-10, and IL-17 in severely ill patients were significantly higher than those in non-severe patients (*P*<0.001, **Figure S3**). In addition, those in the asymptomatic and presymptomatic groups had lower levels of serum IL-6 (median: 1.5 *vs.* 2 pg/ml, *P*-value=0.012, **Table S2**) than patients in the symptomatic group. These findings were consistent with previous reports (21, 22) and suggested that asymptomatic patients have a weaker immune response to SARS-CoV-2 infection than symptomatic patients and play an immune protective role without cytokine storms.

To investigate the association of lymphocyte function with COVID-19 severity, we further analyzed single-cell sequencing data obtained from lung lavage fluids. Compared with that in severe COVID-19 patients, the proportion of lymphocytes (mainly T lymphocytes) increased significantly in mild COVID-19 patients (**Figure 5B**). The T-distribution stochastic neighbor embedding (t-SNE) plot showed that 13 cell clusters were identified from COVID-19 patients (**Figures 5C, D**). CD8+ T cells were subclustered by the known markers of CD3D and CD8A (**Figure 5E**). Furthermore, the inhibitory membrane receptors expressed by CD8+ T cells, such as PDCD1 (PD1), CTLA4, and HAVCR2 (TIM3), were significantly upregulated in severe COVID-19 patients when compared with those in healthy people and mild COVID-19 patients (**Figure 5F**). Surprisingly, the expression of interferon gamma (IFNγ) in severely ill patients was significantly higher than that in non-severely ill patients. To identify the specific inhibitor phenotype related to IFNγ, the co-expression of cytokines and inhibitory membrane receptors was further analyzed. This revealed that the LAG3+IFNγ+CD8+ T cells (10.12% CD8+ T cells in mildly symptomatic COVID-19 patients and 25.83% CD8+ T cells in severely symptomatic COVID-19 patients) and CTLA4+IL10RA+CD8+ T cells (4.42% CD8+ T in mildly symptomatic COVID-19 patients and 13.88% CD8+ T in severely symptomatic COVID-19 patients) might contribute to the T cell exhaustion associated with the severe phenotype of COVID-19 in patients (**Figures 5G–J**).

## Discussion

To control the worldwide pandemic of SARS-CoV-2 infection and efficiently allocate medical resources, the screening and management of asymptomatic or presymptomatic COVID-19 patients in the population are urgent challenges. This retrospective study systematically characterized the asymptomatic, presymptomatic, and symptomatic COVID-19 patients who were admitted to Wuhan Huoshenshan Hospital and developed a risk-stratification calculator that will help clinicians worldwide to make appropriate clinical decisions at the time of admission.

The incubation period for COVID-19, which is defined as the time from exposure to the virus (becoming infected) to symptom onset, varies between individuals. Some people can test positive for COVID-19 from 1-14 days before they develop symptoms (23), with an average of 5-6 days (24). Patients were quarantined as soon as they were considered had exposure to virus, and they were tested for virus upon admission. We counted the time from the first positive nucleic acid test to symptom onset in presymptomatic patients, which was averaged 14.2 days and ranged 1-32 days. There was no difference in the incubation time between non-severe and severe patients in the presymptomatic group (15.78 vs 13.31 days, P-value=0.65).

Asymptomatic and presymptomatic COVID-19 patients have similar CT findings, which indicates the urgent need for a risk-stratification model to differentiate these patients. Despite the absence of validation cohorts to confirm the performance accuracy of the differential diagnosis model, the performance of this risk-stratification model is satisfactory and accurate (based on AUCs >0.82 in both the development and validation cohorts). The package-based prediction calculator can be utilized by clinicians to perform risk stratification of asymptomatic, non-severe presymptomatic, and severe presymptomatic COVID-19 patients based on 5–10 commonly evaluated indicators. This makes the package-based calculator easy to utilize, facilitating clinical resource reallocation, such as for ICU beds and ventilators. Due to the absence of symptoms on admission, the progression of presymptomatic patients did not receive enough attention, while they progressed rapidly probably no timely treatment were given, resulted in even more need for ICU care. These results suggested that presymptomatic patients remain at a higher risk for the ICU if they progress into severe illness. Our model can help predict the disease progression and these patients could avoid getting into the condition that need ICU care.

Furthermore, the risk-stratification model can be utilized for symptomatic and presymptomatic COVID-19 patients, which implies that the indicators identified in the analysis possibly have intrinsic associations with the development of COVID-19. A high BNP level is related to acute respiratory distress syndrome, sepsis, and congestive heart failure and contributes to higher mortality in patients with pneumonia (25, 26). PCT, as an indicator for bacterial coinfection, is approved by the US Food and Drug Administration to guide antibiotic treatment for suspected lower respiratory tract infections (27). Previously published literature reviews and meta-analyses have indicated that the results of laboratory tests for PCT could indicate progression of COVID-19 (8, 28, 29), which greatly supports our results. There is limited information about the frequency and bacterial spectrum of pulmonary coinfections and superinfections in COVID-19 patients (30); however, our analysis strengthens the vital role of BNP and PCT evaluation in the clinic to verify the possibility of coinfections associated with SARS-CoV-2 infection.

Functional CD8+ T cell exhaustion contributes to the progression of SARS-CoV-2 infection (31, 32). As indicated in a previous report, loss of function occurs hierarchically in exhausted CD8+ T cells (33). In the initial stage, CD8+ T cells are unable to produce IL-2. During the intermediate stage, CD8+ T cells may lose the ability to produce tumor necrosis factor (TNF). In the severe stage, CD8+ T cells completely lose the ability to produce large amounts of IFNγ or there may be a physical deletion of virus-specific CD8+ T cells. Based on the single-cell sequencing data, our re-analysis indicates that SARS-CoV-2 can induce intermediate-stage CD8+ T cell exhaustion involving a lack of IL-2 and TNFα production but with limited IFNγ production (**Figure 5F**), which may be mediated by LAG-3. Because LAG-3 cross-linking can inhibit IL-2, TNFα, and IFNγ production and T cell proliferation (34). Therefore, LAG3+IFNγ+CD8+ T cells may be the predominant phenotype showing exhaustion and may need to be further investigated to elucidate the pathogenic clinical severity of COVID-19.

As the retrieved and analyzed clinical data were based on all data from the clinical practice and were not derived from random selection, the data are deemed to be descriptive to some extent. Noteworthy, in this retrospective study, strict criteria for selecting asymptomatic and presymptomatic patients were employed. Although the sample sizes of asymptomatic and presymptomatic patients analyzed in this study are relatively small, it is still a valuable data set analyzed for early COVID-19 patients stratification. In addition, this is a reasonable proportion for both categories evidenced by other studies (7, 15). For a comparable data analysis, baseline normalization by the propensity score matching (PSM) method was performed, and this strategy helped reveal more information from these data. We were unable to verify the accuracy of the model due to lack of data from another research center, resulting in only partial validation of our risk-stratification model.

## Conclusions

This risk-stratification model can facilitate the early prediction of disease progression in asymptomatic, severe presymptomatic, and non-severe presymptomatic patients based on 10 laboratory indicators. It can help make appropriate clinical decisions and optimize the use of limited medical resources. This model and the 10 laboratory indicators can help improve the clinical management of COVID-19 patients who are at different stages of disease evolution.

## Data Availability Statement

Publicly available datasets were analyzed in this study. The datasets used and/or analyzed during the current study are available from the corresponding author on reasonable request.

## Ethics Statement

This study was approved by the Medical Ethical Committee of Wuhan Huoshenshan Hospital (HSSLL011), and the Ethical Committee of Nanjing Medical University (2020-511). Written informed consent was obtained from each patient. Written informed consent to participate in this study was provided by the participants’ legal guardian/next of kin.

## Author Contributions

LS, RD, TZ, and WW contributed equally. YX, SL, PL, and XX had full access to all of the data in the study and takes responsibility for the integrity of the data and the accuracy of the data analysis. Concept and design: YX, SL, PL, and XX. Experiments and data collection: XX. Data analysis and interpretation: SL, RD, TZ, WW, ZW, XJ, KL, YL, JL, MZ, BH (11^th^ author), LW, MW, JC, CW, BH (16^th^ author), HS, CL, and QW. All authors contributed to the article and approved the submitted version.

## Funding

This study was supported by the National Natural Science Foundation of China (Grant No. 81972358, 91959113), Key Foundation of Wuhan Huoshenshan Hospital (Grant No. 2020[18]), Key Research & Development Program of Jiangsu Province (Grant No. BE2017733, BE2018713), Medical Innovation Project of Logistics Service (Grant No. 18JS005), Basic Research Program of Jiangsu Province (Grant No. BK20180036). Novel coronavirus pneumonia technology project of Kunshan first people’s Hospital (XGF202007). Scientific research funds of Sir Run Run Shaw Hospital (Ytp1902).

## Conflict of Interest

The authors declare that the research was conducted in the absence of any commercial or financial relationships that could be construed as a potential conflict of interest.
