# Supplementary material for: Comparative Characterization and Risk Stratification of Asymptomatic and Presymptomatic Patients With COVID-19
Source: Front Immunol. 2021 Jul 9;12:700449. doi: 10.3389/fimmu.2021.700449 (PMC8301218; doi:10.3389/fimmu.2021.700449)
Supplement: Supplementary file 1 [file DataSheet_1.docx]

Supplementary Material

# Supplementary Figures and Tables

## Supplementary Figures


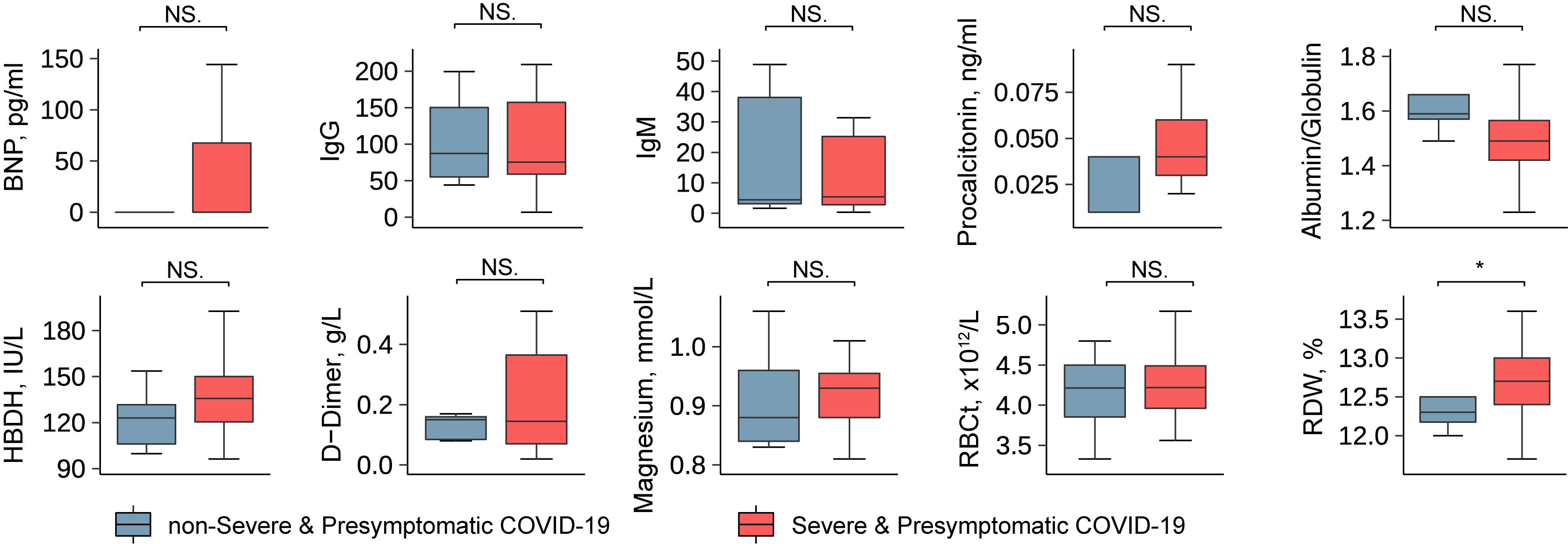


**Supplementary Figure 1.** **A comparison of laboratory values between non-severe and severe presymptomatic patients on admission.** Blue bars represent the non-severe presymptomatic group, and red bars represent severe presymptomatic group, respectively. BNP, Brain Natriuretic Peptide. HBDH, alpha-Hydroxybutyrate Dehydrogenase. RBC, Red Blood Cell Count. RDW, Red Blood Corpuscular Volume Distribution Width. * represents P-value < 0.05. NS. represents P-value > 0.05.


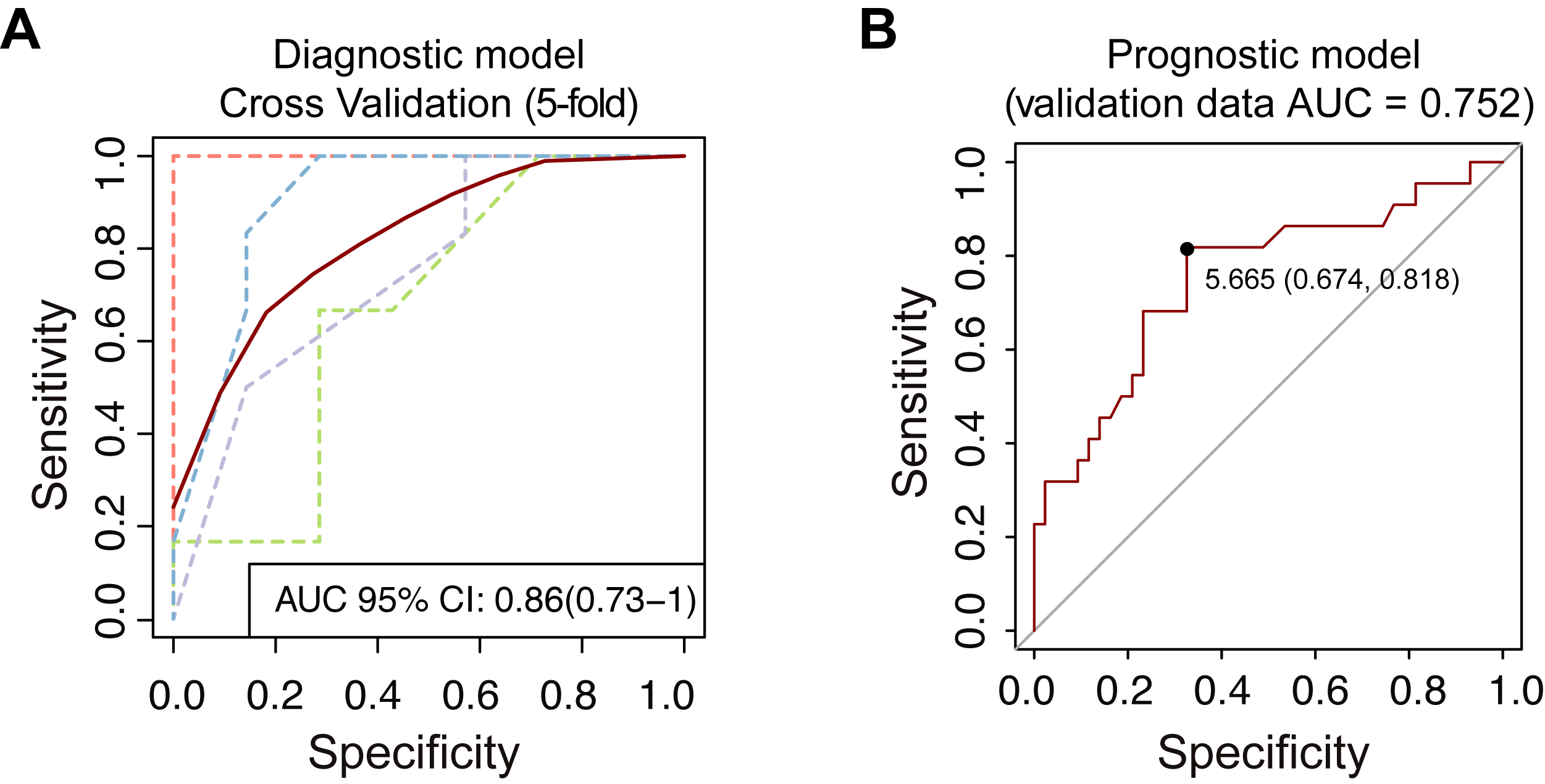


**Supplementary Figure 2.** **A diagnosis and severity prediction model for COVID-19 patients using the top five indicators.** (A) The diagnostic model for asymptomatic and presymptomatic patients. The Receiver Operating Characteristic (ROC) curve of diagnostics of training (red line) and five-fold cross-validation (dotted lines). (B) ROC curve of prognostic of the validation cohort (red lines).


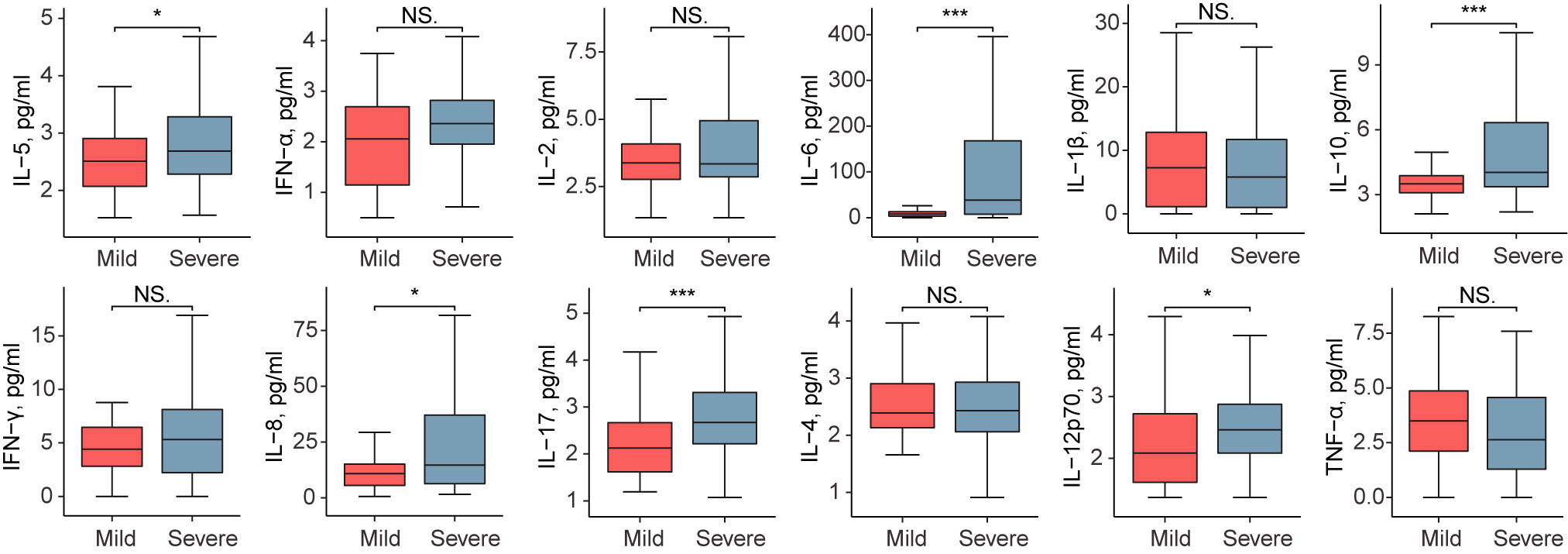


**Supplementary Figure 3.** **Boxplots of the protein levels of plasma cytokines measured by flow** **cytometer without stimulation in symptomatic COVID-19 patients after admission.** The vertical axis indicates the level of protein expression. The *P*-value was calculated using Wilcoxon rank test and shown at the top of each panel. Boxplots represent the 25th and 75th percentiles, with midlines indicating the median values. *** represents *P*-value < 0.001. * represents *P*-value < 0.05. NS. represents *P*-value > 0.05.

## Supplementary Tables

**Table S1. Raw data of clinical characteristics of patients among asymptomatic, presymptomatic and symptomatic COVID-19 before baseline correction.**

|  | **Total (N=2980)** | **Asymptomatic (N=39)** | **Presymptomatic (N=34)** | **Symptomatic (N=2907)** | ***P-*value** |
| --- | --- | --- | --- | --- | --- |
| **Sex– no. (%)** | | | | | 0.743 |
| Male | 1509 (51%) | 22 (56%) | 18 (53%) | 1469 (51%) | - |
| Female | 1471 (49%) | 17 (44%) | 16 (47%) | 1438 (49%) | - |
| **Age (yr.) – median (IQR)** | 60 (50-68) | 53 (44-64) | 57 (39.5-69.5) | 60 (50-68) | 0.031 |
| >50– no. (%) | 2181 (73%) | 22 (56%) | 19 (56%) | 2140 (74%) | 0.005 |
| ≤50– no. (%) | 799 (27%) | 17 (44%) | 15 (44%) | 767 (26%) | - |
| **Comorbidities – no. (%)** | | | | | |
| Hypertension | 911 (31%) | 10 (26%) | 11 (32%) | 890 (31%) | 0.797 |
| Diabetes | 408 (14%) | 7 (18%) | 7 (21%) | 394 (14%) | 0.31 |
| Cardiovascular disease | 338 (11%) | 2 (5%) | 6 (18%) | 330 (11%) | 0.249 |
| Cerebrovascular disease | 119 (4%) | 3 (8%) | 5 (15%) | 111 (4%) | 0.007 |
| Chronic kidney disease | 76 (3%) | 0 (0%) | 0 (0%) | 76 (3%) | 0.845 |
| Chronic liver disease | 145 (5%) | 0 (0%) | 0 (0%) | 145 (5%) | 0.211 |
| **Degree of severity – no. (%)** | | | | | <0.001 |
| Mild/Moderate | 1433 (48%) | 39 (100%) | 9 (26%) | 1385 (48%) | - |
| Severe/Critical | 1547 (52%) | 0 (0%) | 25 (74%) | 1522 (52%) | - |
| **Hospital stays – median (IQR)** | 13 (8-19) | 7 (6-12.5) | 7 (6-15) | 13 (8-19) | <0.001 |
| **Death – no. (%)** | 58 (2%) | 0 (0%) | 1 (3%) | 57 (2%) | 0.579 |
| **Clinical Symptoms – no. (%)** | | | | | |
| Fever | 2178 (73%) | 0 (0%) | 0 (0%) | 2178 (75%) | <0.001 |
| Cough | 2097 (70%) | 0 (0%) | 0 (0%) | 2097 (72%) | <0.001 |
| Shortness of breath | 1320 (44%) | 0 (0%) | 0 (0%) | 1320 (45%) | <0.001 |
| Chest Congestion | 1157 (39%) | 0 (0%) | 0 (0%) | 1157 (40%) | <0.001 |
| Fatigue | 681 (23%) | 0 (0%) | 0 (0%) | 681 (23%) | <0.001 |
| Muscle soreness | 118 (4%) | 0 (0%) | 0 (0%) | 118 (4%) | 0.421 |
| Headache | 18 (1%) | 0 (0%) | 0 (0%) | 18 (1%) | 1 |
| Dizziness | 26 (1%) | 0 (0%) | 0 (0%) | 26 (1%) | 1 |
| Nausea | 21 (1%) | 0 (0%) | 0 (0%) | 21 (1%) | 1 |
| Vomiting | 21 (1%) | 0 (0%) | 0 (0%) | 21 (1%) | 1 |
| Diarrhea | 66 (2%) | 0 (0%) | 0 (0%) | 66 (2%) | 1 |
| Respiratory failure | 36 (1%) | 0 (0%) | 0 (0%) | 36 (1%) | 1 |
| **Viral load of threat swabs (N=1503) – median (IQR)** | | | | | |
| *ORF1ab* | 37.41 (34.27-39.82) | 41.21 (39.48-42.06) | 37 (35.93-39.45) | 37.41 (34.24-39.8) | 0.203 |
| *N* | 35.98 (33.02-37.53) | 36.59 (36.12-37.07) | 36.26 (35.12-37.27) | 35.96 (33-37.53) | 0.768 |
| **Anti-SARS-Cov-2 IgG and IgM level – median (IQR)** | | | | | |
| IgG level | 140.11 (75.66-180.56) | 61.66 (9.5-120.75) | 87.69 (52.71-119.35) | 142.39 (76.96-181.19) | <0.001 |
| IgM level | 26.41 (7.75-66.79) | 4.49 (1.11-39.34) | 5.04 (2.53-17.27) | 27.06 (8.15-67.26) | <0.001 |

Data are median (IQR), no. or N (%), where N is the total number of patients with available data. P-values comparing asymptomatic, presymptomatic and symptomatic cases are from Fisher’s exact test, or Mann-Whitney U test.

**Table S2. Laboratory findings of patients among asymptomatic, presymptomatic and symptomatic COVID-19.**

|  | **Normal Range** | **Total** | **Asymptomatic** | **Presymptomatic** | **Symptomatic** | ***P*-value** |
| --- | --- | --- | --- | --- | --- | --- |
| **Heart injury markers – median (IQR)** | | | | | | |
| Brain natriuretic peptide, pg/ml | 0-100 | 0.01 (0.01-46.5) | 0.01 (0.01-14.48) | 0.01 (0.01-176.38) | 0.01 (0.01-46.57) | 0.174 |
| Creatine kinase, IU/L | 24-190 | 43.6 (29.5-65.9) | 56.8 (33.97-80.85) | 68.1 (44.7-112.45) | 43.3 (29.25-65.15) | <0.001 |
| Creatine kinase isoenzyme, IU/L | 0-24 | 8.4 (6.8-11) | 8.6 (7.03-10.28) | 10.2 (8.55-12.1) | 8.4 (6.7-10.9) | 0.002 |
| Lactate dehydrogenase, IU/L | 120-250 | 179.8 (153.1-220.8) | 159.75 (143.27-184.3) | 161.1 (139.28-190.57) | 180.4 (153.5-221.9) | <0.001 |
| α- hydroxybutyrate dehydrogenase, IU/L | 72-182 | 145.2 (124.4-179.2) | 126.4 (115.4-144.4) | 130.55(119.35-152.35) | 145.7 (124.8-179.8) | <0.001 |
| **Liver injury markers – median (IQR)** | | | | | | |
| Alanine transaminase, IU/L | 9-50 | 25.1 (15.8-43.5) | 13.2 (11.3-38) | 19.8 (13.75-27.95) | 25.5 (16-43.6) | <0.001 |
| Aspartate Transaminase, IU/L | 9-60 | 20.1 (15.7-28.1) | 17.8 (13.8-26) | 19.05 (15.88-25.6) | 20.1 (15.7-28.2) | 0.237 |
| Total protein, g/L | 65-85 | 64.8 (60.4-68.7) | 67.5 (63.5-71.1) | 67.5 (64.55-70.05) | 64.8 (60.3-68.7) | <0.001 |
| Albumin g/L | 40-55 | 37.8 (34.8-40.5) | 40.3 (38.3-42.9) | 40.3 (37.95-42.1) | 37.7 (34.7-40.4) | <0.001 |
| γ-glutamyl transpeptidase, IU/L | 10-60 | 33 (21.3-54.75) | 22.6 (14.5-48.1) | 27.2 (18.5-42.55) | 33.3 (21.4-55.2) | <0.001 |
| **Kidney injury marker – median (IQR)** | | | | | | |
| Glucose, mmol/L | 3.9-6.1 | 5.05 (4.54-6.53) | 4.84 (4.46-5.44) | 4.65 (4.15-5.79) | 5.05 (4.55-6.57) | 0.003 |
| Urea nitrogen, umol/L | 3.6-9.5 | 4.63 (3.74-5.8) | 4.28 (3.61-5.13) | 5.04 (3.99-6.85) | 4.63 (3.75-5.79) | 0.0424 |
| Uric acid, umol/L | 202-416 | 281 (221-345.25) | 316.5 (246-360) | 320 (245.5-376) | 280 (220-344) | 0.019 |
| Cystatin C, mg/L | 0.63-1.25 | 0.94 (0.83-1.09) | 0.84 (0.78-0.97) | 0.92 (0.81-1.3) | 0.94 (0.83-1.09) | 0.006 |
| **Coagulation function – median (IQR)** | | | | | | |
| D-Dimer, g/L | 0-0.55 | 0.44 (0.22-1.04) | 0.22 (0.16-0.33) | 0.15 (0.09-0.39) | 0.46 (0.23-1.06) | <0.001 |
| Plasma fibrinogen, g/L | 2-4 | 2.93 (2.58-3.37) | 2.74 (2.41-3.27) | 2.62 (2.37-3.04) | 2.93 (2.59-3.38) | 0.002 |
| Thrombin time, s | 10-20 | 15.16 (14.44-15.96) | 14.61 (14.32-15.48) | 14.8 (14.45-15.57) | 15.18 (14.45-15.96) | 0.044 |
| **Infection-related markers – median (IQR)** | | | | | | |
| Interleukin-6, pg/ml | 0-7 | 2 (1.5-4.39) | 1.5 (1.5-2.04) | 1.5 (1.5-2.82) | 2 (1.5-4.53) | 0.012 |
| C-reactive protein, mg/L | 0-4 | 2.2 (0.79-8.4) | 1.15 (0.48-4.63) | 1.96 (0.28-5.24) | 2.21 (0.82-8.61) | 0.001 |
| Hypersensitive C - reactive protein, mg/L | 0-4 | 2.21 (0.79-8.4) | 1.15 (0.48-4.63) | 1.96 (0.28-5.24) | 2.22 (0.82-8.61) | 0.002 |
| Procalcitonin, ng/ml | 0-0.05 | 0.04 (0.03-0.07) | 0.03 (0.03-0.05) | 0.04 (0.03-0.09) | 0.04 (0.03-0.07) | 0.041 |
| **Blood routine examination – median (IQR)** | | | | | | |
| Lymphocyte percentage, % | 20-50 | 26.2 (18.78-32.7) | 29.7 (21.38-35.3) | 27.45 (22.9-34.4) | 26.1 (18.6-32.6) | 0.009 |
| Lymphocyte, x10^9^/L | 1.1-3.2 | 1.48 (1.07-1.89) | 1.73 (1.38-2.06) | 1.62 (1.13-1.95) | 1.47 (1.06-1.88) | 0.004 |
| Monocyte percentage, % | 3-10 | 7.5 (6.1-9) | 6.55 (6-7.72) | 7.15 (6.07-8.4) | 7.5 (6.1-9) | 0.011 |
| Monocyte, x10^9^/L | 0.1-0.6 | 0.44 (0.35-0.56) | 0.4 (0.34-0.47) | 0.42 (0.34-0.46) | 0.44 (0.35-0.57) | 0.005 |
| Basophil percentage, % | 0-1 | 0.4 (0.2-0.5) | 0.5 (0.3-0.6) | 0.4 (0.27-0.6) | 0.4 (0.2-0.5) | 0.002 |
| Basophil, x10^9^/L | 0-0.06 | 0.02 (0.01-0.03) | 0.03 (0.02-0.04) | 0.02 (0.02-0.03) | 0.02 (0.01-0.03) | 0.002 |
| Red blood cell count, x10^12^/L | 4.3-5.8 | 4.01 (3.64-4.36) | 4.23 (3.87-4.61) | 4.12 (3.61-4.43) | 4.01 (3.64-4.36) | 0.004 |
| Red blood corpuscular volume distribution width, % | 10.9-15.4 | 13 (12.5-13.7) | 12.7 (12.3-13.38) | 12.75 (12.4-13.43) | 13 (12.5-13.7) | 0.038 |
| Mean corpuscular volume | 82-100 | 92.5 (89.8-95.1) | 90.55 (88.73-93.3) | 93.1 (90.2-94.7) | 92.5 (89.82-95.1) | 0.005 |
| Hemoglobin, g/L | 130-175 | 123 (110-135) | 129 (114.25-147.5) | 126 (112-140) | 123 (110-135) | 0.014 |
| **Blood biochemical examination – median (IQR)** | | | | | | |
| Albumin/Globulin | 1-2.4 | 1.41 (1.25-1.56) | 1.52 (1.42-1.68) | 1.49 (1.34-1.59) | 1.41 (1.24-1.56) | <0.001 |
| Phosphorus, mmol/L | 0.85-1.51 | 1.14 (0.99-1.29) | 1.27 (1.11-1.38) | 1.23 (1.15-1.37) | 1.14 (0.99-1.28) | <0.001 |
| Magnesium, mmol/L | 0.75-1.02 | 0.9 (0.85-0.95) | 0.89 (0.84-0.95) | 0.95 (0.87-1) | 0.9 (0.85-0.95) | 0.036 |
| Calcium, mmol/L | 2.11-2.52 | 2.18 (2.09-2.26) | 2.24 (2.18-2.32) | 2.23 (2.18-2.3) | 2.18 (2.09-2.26) | <0.001 |

Data are median (IQR). P-values comparing asymptomatic, presymptomatic and symptomatic cases are from Mann-Whitney U test.

**Table S3. Characteristics of patients between non-Severe and Severe COVID-19 patients.**

|  | **Normal Range** | **Asymptomatic**  **& non-Severe** | **Pre-symptomatic**  **& non-Severe** | **Pre-symptomatic**  **& Severe** | ***P*-value** | **Symptomatic**  **& non-Severe** | **Symptomatic**  **& Severe** | ***P*-value** |
| --- | --- | --- | --- | --- | --- | --- | --- | --- |
| **Viral load of threat swabs – median (IQR)** | | | | | | | | |
| **ORF1ab** | >40 | 41.21 (39.48-42.06) | 34.44 (33.33-38.81) | 37.1 (36.91-38.61) | 0.456 | 37.97 (35.47-40.07) | 37.02 (33.41-39.05) | 0.003 |
| **N** | >40 | 36.59 (36.12-37.07) | 36.32 (32.8-37.26) | 36.26 (35.55-37.59) | 0.617 | 36.46 (34.3-37.8) | 35.66 (32.18-37.11) | <0.001 |
| **Anti-SARS-Cov-2 IgG and IgM level – median (IQR)** | | | | | | | | |
| **IgG level** | 0-10 | 61.66 (9.5-120.75) | 118.7 (55.04-174.71) | 87.05 (52.71-103.19) | 0.42 | 146.9 (79.07-182.37) | 137.81 (77.38-179.77) | 0.155 |
| **IgM level** | 0-10 | 4.49 (1.11-39.34) | 3.07 (2.69-5.68) | 6.13 (2.51-19.93) | 0.663 | 29.27 (10.86-66.8) | 27 (8.01-78.46) | 0.514 |
| **Heart injury markers – median (IQR)** | | | | | | | | |
| Brain natriuretic peptide, pg/ml | 0-100 | 0.01 (0.01-14.48) | 0.01 (0.01-0.01) | 33.14 (0.01-208.56) | 0.197 | 0.01 (0.01-10.97) | 17.04 (0.01-87.4) | <0.001 |
| Creatine kinase, IU/L | 24-190 | 56.8 (33.97-80.85) | 66 (51.2-74.3) | 68.1 (45.2-115.6) | 0.693 | 46.5 (33.52-67) | 41.2 (26.6-63.3) | <0.001 |
| Creatine kinase isoenzyme, IU/L | 0-24 | 8.6 (7.03-10.28) | 7.2 (6.95-11.6) | 10.2 (8.6-12.1) | 0.174 | 7.9 (6.5-9.8) | 8.8 (6.9-11.9) | <0.001 |
| Lactate dehydrogenase, IU/L | 120-250 | 159.75 (143.27-184.3) | 155.3 (130.25-164.25) | 161.1 (142.1-194.6) | 0.090 | 165.7 (146.4-194.9) | 192.7 (162.9-241.3) | <0.001 |
| α- hydroxybutyrate dehydrogenase, IU/L | 72-182 | 126.4 (115.4-144.4) | 123 (106.1-131.65) | 135.8 (121-156.5) | 0.125 | 134 (118.25-156.67) | 154.5 (131.6-199) | <0.001 |
| **Liver injury markers – median (IQR)** | | | | | | | | |
| Aspartate Transaminase, IU/L | 9-60 | 17.8 (13.8-26) | 16 (15.28-16.47) | 21.15 (16.1-25.95) | 0.030 | 19.4 (15.4-26.48) | 20.5 (15.9-30) | <0.001 |
| Total protein, g/L | 65-85 | 67.5 (63.5-71.1) | 66.9 (65.38-68.1) | 67.5 (64.4-70.85) | 0.746 | 65.5 (61.9-69) | 64.1 (59.25-68.45) | <0.001 |
| Albumin g/L | 40-55 | 40.3 (38.3-42.9) | 41.25 (40.75-41.7) | 40.1 (36.6-42.55) | 0.218 | 38.6 (36.3-41) | 37 (33.3-39.9) | <0.001 |
| total bilirubin, umol/L | 0-26 | 9.5 (7.5-12.2) | 7.35 (7.18-9.47) | 9.4 (6.45-11.35) | 0.569 | 8.9 (7-11.7) | 9.4 (7.1-12.3) | 0.003 |
| Direct Bilirubin, umol/L | 0-8 | 3.3 (2.7-3.9) | 2.95 (2.63-3.12) | 3.1 (2.6-4.4) | 0.312 | 3 (2.4-3.95) | 3.2 (2.4-4.6) | <0.001 |
| Total bile acid, umol/L | 0-10 | 4 (2.5-5.9) | 3.95 (2.62-4.78) | 4.6 (2.7-6.1) | 0.413 | 3.7 (2.4-5.9) | 4.2 (2.6-6.9) | <0.001 |
| Alkaline phosphatase, IU/L | 45-125 | 64.3 (57.7-89.7) | 72.3 (60.75-78.88) | 76.7 (60.25-93.75) | 0.223 | 70.3 (58.3-84.1) | 72.85 (59.2-90.1) | <0.001 |
| γ-glutamyl transpeptidase, IU/L | 10-60 | 22.6 (14.5-48.1) | 16.05 (12.57-26.68) | 28.4 (21.2-44.5) | 0.023 | 31.65 (20.3-51.88) | 34.15 (22.33-56.48) | 0.002 |
| **Kidney injury markers – median (IQR)** | | | | | | | | |
| Glucose, mmol/L | 3.9-6.1 | 4.84 (4.46-5.44) | 4.61 (4.46-5.04) | 4.8 (4.15-5.79) | 0.889 | 4.8 (4.43-5.54) | 5.3 (4.68-7.25) | <0.001 |
| Urea nitrogen, umol/L | 3.6-9.5 | 4.28 (3.61-5.13) | 4.77 (4.26-5.02) | 5.34 (3.99-7.17) | 0.14 | 4.31 (3.6-5.26) | 4.88 (3.9-6.34) | <0.001 |
| Uric acid, umol/L | 202-416 | 316.5 (246-360) | 278 (236.75-347.75) | 321 (248.5-376) | 0.613 | 293 (241-351) | 270 (206-338) | <0.001 |
| Cystatin C, mg/L | 0.63-1.25 | 0.84 (0.78-0.97) | 0.74 (0.73-0.85) | 1 (0.83-1.5) | 0.018 | 0.89 (0.8-1.01) | 0.98 (0.86-1.17) | <0.001 |
| Carbon dioxide, mmol/L | 22-29 | 24.55 (23.15-25.8) | 24.7 (23.18-25.33) | 24.5 (22.7-25.8) | 0.928 | 24.2 (22.8-25.6) (862) | 24.5 (22.9-26.1) | 0.001 |
| **Coagulation function – median (IQR)** | | | | | | | | |
| D-Dimer, g/L | 0-0.55 | 0.22 (0.16-0.33) | 0.15 (0.08-0.16) | 0.16 (0.09-0.42) | 0.522 | 0.3 (0.16-0.53) | 0.62 (0.31-1.49) | <0.001 |
| Plasma fibrinogen, g/L | 2-4 | 2.74 (2.41-3.27) | 2.54 (2.51-2.7) | 2.63 (2.36-3.05) | 0.927 | 2.89 (2.57-3.24) | 2.97 (2.6-3.45) | <0.001 |
| International normalized ratio | 0.8-1.25 | 1.06 (1-1.11) | 1.11 (1.11-1.15) | 1.11 (1.01-1.21) | 0.68 | 1.06 (1.02-1.12) | 1.08 (1.02-1.16) | <0.001 |
| PT-% | 70-125 | 97.45 (92.97-100.38) | 93.1 (90.7-96.9) | 93.4 (87.7-99.3) | 0.909 | 96.2 (93-99.2) | 95 (90.3-98.6) | <0.001 |
| Prothrombin time, s | 9.2-15 | 12.62 (11.98-13.38) | 13.33 (12.59-13.85) | 13.27 (12.16-14.52) | 0.731 | 12.74 (12.2-13.39) | 12.97 (12.29-13.93) | <0.001 |
| Thrombin time, s | 10-20 | 14.61 (14.32-15.48) | 14.52 (14.26-14.57) | 15.04 (14.46-15.62) | 0.163 | 15.05 (14.38-15.79) | 15.25 (14.49-16.07) | <0.001 |
| **Infection-related markers – median (IQR)** | | | | | | | | |
| Interleukin-6, pg/ml | 0-7 | 1.5 (1.5-2.04) | 1.5 (1.5-1.5) | 1.5 (1.5-5.77) | 0.148 | 1.63 (1.5-3.29) | 2.04 (1.5-5.73) | <0.001 |
| C-reactive protein, mg/L | 0-4 | 1.15 (0.48-4.63) | 1.01 (0.18-2.35) | 2.1 (0.33-5.3) | 0.159 | 1.53 (0.62-4.04) | 2.97 (0.96-15.13) | <0.001 |
| Hypersensitive C - reactive protein, mg/L | 0-4 | 1.15 (0.48-4.63) | 1.01 (0.18-2.35) | 2.1 (0.33-5.3) | 0.159 | 1.53 (0.63-4.07) | 2.97 (0.96-10) | <0.001 |
| Procalcitonin, ng/ml | 0-0.05 | 0.03 (0.03-0.05) | 0.01 (0.01-0.04) | 0.05 (0.03-0.09) | 0.012 | 0.04 (0.03-0.05) | 0.05 (0.03-0.1) | <0.001 |
| **Blood routine examination – median (IQR)** | | | | | | | | |
| Lymphocyte percentage, % | 20-50 | 29.7 (21.38-35.3) | 29.9 (28.38-32.92) | 25.8 (22.2-34.4) | 0.219 | 29.2 (23.3-34.5) | 24 (15-31.33) | <0.001 |
| Lymphocyte, x10^9^/L | 1.1-3.2 | 1.73 (1.38-2.06) | 1.86 (1.66-2.14) | 1.52 (1.11-1.94) | 0.122 | 1.59 (1.25-2) | 1.37 (0.94-1.82) | <0.001 |
| Neutrophil percentage, % | 40-75 | 59.65 (53.9-67.2) | 59.35 (58.82-60.85) | 64.35 (55.35-67.35) | 0.298 | 59.9 (54.1-66.2) | 64.7 (56.8-75.23) | <0.001 |
| Neutrophil, x10^9^/L | 1.8-6.3 | 3.65 (2.69-4.34) | 3.58 (2.89-4.56) | 3.38 (2.76-4.37) | 0.96 | 3.34 (2.58-4.33) | 3.91 (2.83-5.71) | <0.001 |
| Monocyte, x10^9^/L | 0.1-0.6 | 0.4 (0.34-0.47) | 0.47 (0.39-0.54) | 0.42 (0.34-0.46) | 0.177 | 0.43 (0.34-0.53) | 0.46 (0.36-0.59) | <0.001 |
| Basophil percentage, % | 0-1 | 0.5 (0.3-0.6) | 0.3 (0.3-0.55) | 0.4 (0.2-0.57) | 0.96 | 0.4 (0.2-0.6) | 0.3 (0.2-0.5) | <0.001 |
| Basophil, x10^9^/L | 0-0.06 | 0.03 (0.02-0.04) | 0.02 (0.01-0.03) | 0.02 (0.02-0.03) | 0.984 | 0.02 (0.01-0.03) | 0.02 (0.01-0.03) | <0.001 |
| Eosinophil percentage, % | 0.4-8 | 2.1 (1.5-3.22) | 2 (1.65-2.83) | 1.95 (1.33-2.8) | 0.706 | 2.1 (1.3-3.4) | 1.9 (1-3.4) | <0.001 |
| White blood cell count, x10^9^/L | 3.5-9.5 | 5.85 (5.1-7.07) | 6.1 (5.17-7.28) | 5.7 (5-6.4) | 0.487 | 5.6 (4.6-6.9) | 6.2 (4.9-8.1) | <0.001 |
| Red blood cell count, x10^12^/L | 4.3-5.8 | 4.23 (3.87-4.61) | 4.17 (4.02-4.44) | 4.07 (3.6-4.42) | 0.41 | 4.15 (3.81-4.47) | 3.91 (3.48-4.26) | <0.001 |
| Red blood corpuscular volume distribution width, % | 10.9-15.4 | 12.7 (12.3-13.38) | 12.45 (12.2-12.5) | 13 (12.5-13.5) | 0.003 | 12.8 (12.3-13.4) | 13.2 (12.6-14) | <0.001 |
| Hematocrit | 40-50 | 38.15 (34.4-43.25) | 37.5 (35.35-40.25) | 37.65 (33.2-41.1) | 0.758 | 38.1 (35.2-41) | 36 (32.5-39.1) | <0.001 |
| Mean corpuscular volume | 82-100 | 90.55 (88.73-93.3) | 90.4 (89.35-93.7) | 93.35 (90.62-94.7) | 0.23 | 92.1 (89.7-94.5) | 92.8 (89.9-95.6) | <0.001 |
| Mean platelet volume | 8-10 | 9.9 (9.3-11.07) | 10.25 (9.95-10.83) | 9.8 (8.85-11.4) | 0.721 | 9.9 (9.3-10.7) | 10 (9.3-10.8) | 0.012 |
| Platelet count, x10^9^/L | 125-350 | 223.5 (189.25-274) | 236.5 (229-248.5) | 217 (156-289.75) | 0.766 | 222 (185-273) | 216 (166-269) | <0.001 |
| Hemoglobin, g/L | 130-175 | 129 (114.25-147.5) | 128.5 (120-139.5) | 126 (108.5-138.5) | 0.483 | 129 (118-139) | 119 (104-131) | <0.001 |
| Mean corpuscular hemoglobin concentration, g/L | 316-354 | 338 (332-342) | 343 (338.5-347.5) | 337.5 (329-343) | 0.031 | 338 (333-342) | 335 (329-340) | <0.001 |
| **Blood biochemical examination – median (IQR)** | | | | | | | | |
| Albumin/Globulin | 1-2.4 | 1.52 (1.42-1.68) | 1.59 (1.57-1.66) | 1.48 (1.32-1.54) | 0.032 | 1.45 (1.32-1.58) | 1.37 (1.19-1.54) | <0.001 |
| Phosphorus, mmol/L | 0.85-1.51 | 1.27 (1.11-1.38) | 1.33 (1.31-1.35) | 1.21 (1.13-1.38) | 0.437 | 1.16 (1.03-1.29) | 1.12 (0.96-1.27) | <0.001 |
| Potassium, mmol/L | 3.5-5.3 | 4.21 (4.05-4.61) | 4.24 (4.06-4.55) | 4.41 (4.21-4.7) | 0.306 | 4.36 (4.09-4.63) | 4.26 (3.96-4.58) | <0.001 |
| Sodium, mmol/L | 137-147 | 141.45 (139.8-142.43) | 142.85 (141.6-143.7) | 140.25 (138.12-142.12) | 0.084 | 141.5 (140.1-143) | 141.2 (139.2-143.1) | <0.001 |
| Chlorine, mmol/L | 99-110 | 106.35 (104.47-107.5) | 107.2 (106.63-107.68) | 104.75 (103.1-107.88) | 0.103 | 106.2 (104.5-107.9) | 105.5 (103.1-107.5) | <0.001 |
| Calcium, mmol/L | 2.11-2.52 | 2.24 (2.18-2.32) | 2.26 (2.23-2.28) | 2.22 (2.18-2.31) | 0.441 | 2.19 (2.12-2.26) | 2.17 (2.07-2.27) | <0.001 |

Data are median (IQR). P-values comparing non-severe and severe cases are from Wilcoxon rank test in presymptomatic and symptomatic group, respectively.
